# Measuring the Electromagnetic Field of the Human Brain at a Distance Using a Shielded Electromagnetic Field Channel

**DOI:** 10.7759/cureus.23626

**Published:** 2022-03-29

**Authors:** James Brazdzionis, James Wiginton, Tye Patchana, Paras Savla, James Hung, Yongming Zhang, Dan E Miulli

**Affiliations:** 1 Neurosurgery, Riverside University Health System Medical Center, Moreno Valley, USA; 2 Electrical Engineering, Quasar Federal Systems, San Diego, USA; 3 Medical Physics, Quasar Federal Systems, San Diego, USA; 4 Neurosurgery, Arrowhead Regional Medical Center, Colton, USA

**Keywords:** neuroscience, magnetic field sensing, helmet, neurosurgery, technology, electromagnetic field

## Abstract

Introduction

The electromagnetic field (EMF) of the human brain generated by the movement of ions in the brain can be measured in a novel manner. The measurement can be completed through the skull, in a non-contact, non-invasive, continuous manner using a lightweight helmet. This investigation was conducted to determine if brain activity from movement and thoughts of movement can be measured at a distance and if that measurement can be readily evaluated at a distance using shielding with a shielded helmet and a shielded EMF channel surrounding a sensor.

Methods

Non-clinical human subject volunteers donned a lightweight sensor helmet and performed a variety of specific tasks synchronized with an audible tone generated by a metronome. Constructs were created to determine if the human subjects’ brain EMF can be recorded at a distance using sensors surrounded by shielding acting similar to a waveguide in an EMF channel connected to a shielded helmet.

Results

The EMF sensors appeared to record brain electromagnetic activity as it is funneled into a shielded channel acting as a waveguide at a considerable distance including distances as far as 63 cm away.

Conclusion

Specific brain EMFs from movement, thoughts of movement, and emotional thought can be continuously measured in a non-contact fashion at a distance using an EMF waveguide approach with an EMF channel and shielded helmet.

## Introduction

The nervous system and cortex function through a balance of electrochemical gradients and generation of an electrical current traveling over distances through the balance of competing action potentials, excitatory postsynaptic potentials (EPSPs), and inhibitory postsynaptic potentials (IPSPs) [1]. Action potentials are more short-lived compared to EPSPs and IPSPs. The balance and interactions of these potentials generated by multiple neurons allow for summation of these IPSPs and EPSPs and allow higher level activities [1]. The summation of these potentials is affected by neuronal structure, interactions within the dendritic tree, and orientation, and acts linearly and non-linearly [1,2].

Due to the generation of electromagnetic fields (EMFs) by cortical tissue and other tissues such as the heart, which also generate intrinsic electrical current through balance of their own intrinsic firing, sensors sensitive enough to sense small potentials and potential differences were necessary. Moreover, appropriate shielding to reduce external magnetic interference was paramount. Metallic shielding created with Mu-metal sheets was used in these trials to block any potential outside EMF interference and was previously determined to be functional within these parameters by Wiginton et al. and Brazdzionis et al. [3-5]. Mu-metal is a ferromagnetic alloy made of nickel-iron that is frequently used for shielding electronics against magnetic fields due to high magnetic permeability, allowing for absorption of magnetic energy [6].

It has been demonstrated that the brain’s EMF can be measured in a non-invasive fashion using proprietary sensors [3]. It was further demonstrated that stereotyped tasks can be measured and interpreted using these same sensors [4]. The current study wished to investigate if there was an optimal set distance from the skull to measure these action potentials as well as to identify if there was a functional limitation to measuring these potentials at a distance using a shielded helmet in a shielded room.

## Materials and methods

This study was approved by our institution's institutional review board. Proprietary induction sensors (patented, Model BS-1000, Quasar Federal Systems, San Diego, CA) were used to measure the EMF of specific human brain activities in a non-contact and non-invasive fashion using a prototype lightweight helmet as previously described by Wiginton et al. and Brazdzionis et al. [3,4]. This helmet was constructed using two layers of Mu-metal (MuMETAL®, Magnetic Shield Corporation, Bensenville, IL) with inner and outer layers of interlaced copper mesh to absorb and reflect the external EMF signal. Both layers of Mu-metal were separated approximately 2.5 cm. Four holes were drilled within the helmet to accommodate four 18-inch induction sensors. These four sensors (Bx, By, Bz, and B319) were configured to bitemporal and bifrontal locations as previously described [3-5]. These induction sensors require a 9-volt power supply and consume 3.3 mA in order to measure non-direct current, low-magnetic fields in a single axis between 1 Hz and 30 kHz through the use of a solenoid coil around a-0.2 inch diameter high-permeability core.

**Figure 1:**
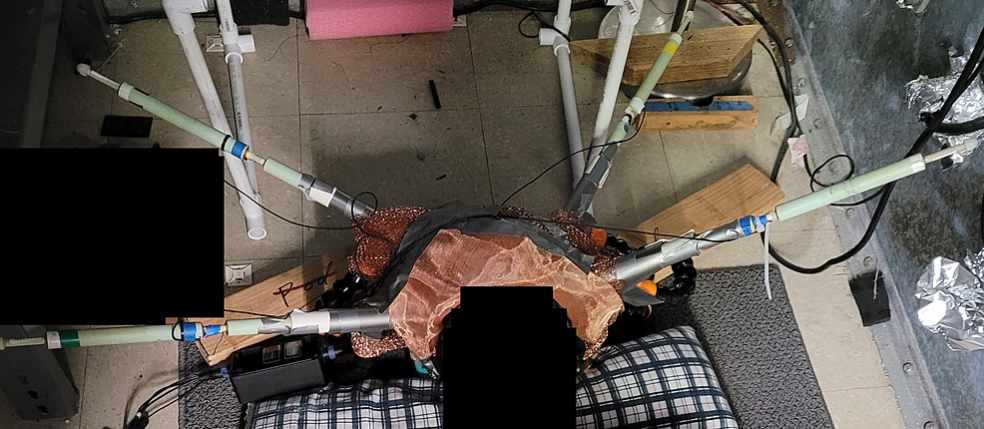
Subject with a helmet in place A subject with the shielded helmet is seen in the image. Sensors Bx, By, Bz, and B319 are in place extending through the electromagnetic field channels (silver metallic extension) extending from the copper-colored helmet.

Each sensor was ensured to be positioned with the positive end (+) oriented toward the scalp. Furthermore, the Bz sensor was placed on the right side at approximately the right motor strip with the trajectory pointed toward the left motor strip, the By sensor was placed at the right temporal region with a trajectory oriented toward the left motor strip, the B319 sensor was placed on the left side at approximately the location of the left motor strip with a trajectory orienting toward the left motor strip, and the Bx sensor was placed in the left temporal region with a trajectory pointed toward the left motor strip. Each sensor was placed within plastic polyvinyl chloride (PVC) piping. The PVC piping containing the sensor was then wrapped in Mu-metal to form an EMF channel to function similarly to a waveguide in order to focus the generated EMF by targeted brain tissue and exclude external EMF signal. Sensor recordings were recorded on a laptop and post-processed as described by Wiginton et al. using fast Fourier transformation (FFT) [3]. Detection sensitivity for sensors was set at 1.5 pTrms/rtHz at 1 Hz, 0.15 pTrms/rtHz at 10 Hz, 0.025 pTrms/rtHz at 100 Hz, and 0.02 pTrms/rtHz at 1 kHz and above. Sensor responses were captured between 1 Hz and 2 kHz, and a gain filter module was used set with a 10x gain for amplifying signal with a 2-kHz low-pass filter for anti-aliasing. Data capture was completed at 5 kilosamples per second. A subject with the helmet in place can be seen in Figure 1.

**Figure 2:**
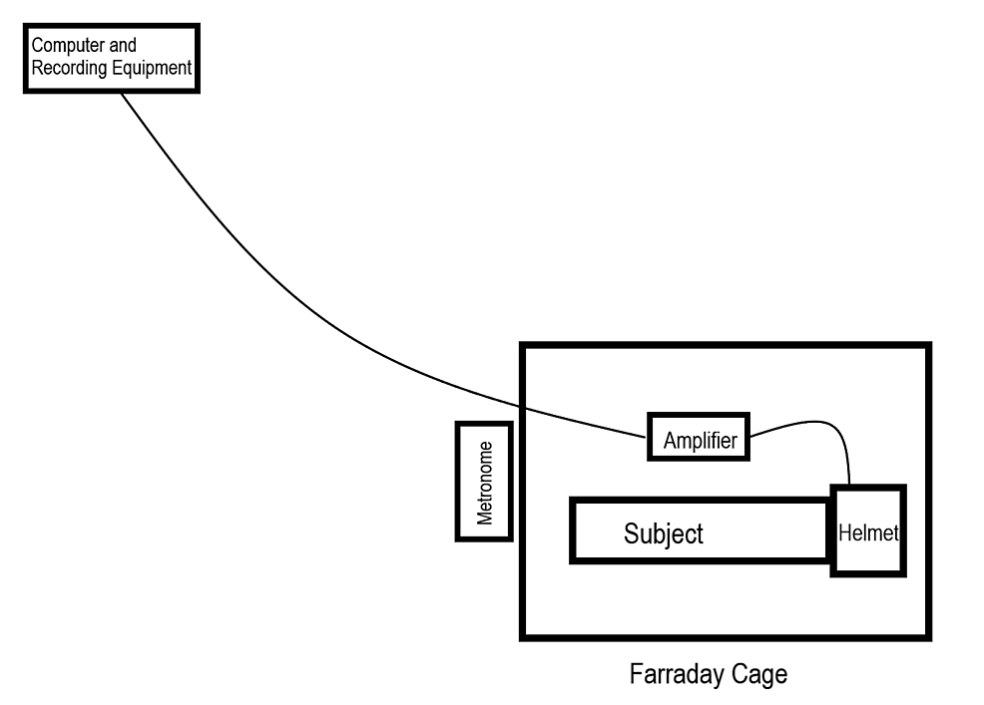
Diagram of a subject within the recording setup Diagram is not drawn to scale. Within the area labeled "Helmet," electromagnetic field channels were connected to the helmet construct with the sensors placed within each channel. Thin lines connecting the helmet to the amplifier and gain filter module and from the amplifier to the computer and recording equipment represent wires connecting the devices.

Each test was conducted with a non-ferromagnetic metronome placed outside the Faraday cage containing the human participant, helmet, and sensors. This metronome produced a sound at 120 bpm (2 Hz) to synchronize activities and in order to detect a reproducible recording of brain activity from each subject. A diagram of the recording (not to scale) setup can be seen in Figure 2.

Volunteer human subjects performed each test by participating in designated reproduced activities. Subjects were placed supine and donned the helmet. Activities involving hand or foot tapping were completed at a rate of 2 Hz. Of note, within the Farraday cage, subjects were asked to keep their eyes closed to limit visual stimulus. Furthermore, the shielded room and Farraday cage were dark to limit visual stimulation. Mechanistically, hand tapping was completed through the protocol delineated by Brazdzionis et al. [4]. This tapping activity entailed lifting the hand as a singular unit while leaving the wrist fixed on the ground. The tips of the fingers were elevated 6 cm and then brought back to the ground. Each elevation and return to baseline consisted of a single tap. Foot tapping was completed with the knees and hips flexed. Each foot was dorsiflexed off the ground with the heel remaining fixed on the ground. Toes were elevated 6 inches through dorsiflexion and plantarflexed back to the ground. Each dorsiflexion and plantar flexion cycle was designated as a single tap. Each test started with 30 seconds of "rest" wherein the participant was asked to not tap and try to maintain a lack of higher cortical thoughts. This rest was followed by trials of 60 seconds of the designated activities with 30-second rest "breaks" between each 60-second activity bin. For analysis, summative FFT transformed data placed into 20-second bins were plotted graphically.

Testing was completed to investigate the ability and the effects of brain attenuation of external magnetic fields on stereotyped motor activities. This was done to assess the effectiveness of measurement of the brain's generated magnetic field and the effects of external magnetic field transmission of the generated field. Further investigation included evaluating sensors from a distance when using Mu-metal shielding as an EMF channel functioning similar to a waveguide to evaluate the decay of the magnetic field over distance.

Effects of externally generated magnetic fields: battery testing

Initial testing was conducted to evaluate the effects of attenuation of external EMF on the brain’s ability to generate EMF and on the ability of the sensor to identify the brain’s EMF with an EMF stimulus in proximity. Subjects were asked to lie supine in a Faraday cage and participate in reproduced tapping activities at 2 Hz. While doing so, the subject was given a 3-volt battery connected to a light-emitting diode (LED). The circuit was created by emitting a light, which resulted in completing a circuit. This circuit was placed in a sealed plastic bag. The active electrical current generated by the LED and battery construct definitionally generates an EMF that must propagate through space to surrounding structures. The subject was asked to hold the active battery and light circuit and participate in the previously described activity of right hand tapping at 2 Hz with the protocol described above and initially described by Brazdzionis et al. [4]. An additional battery was added to the circuit to increase the voltage and current. After initial testing, the subject placed the battery and light constructs in a plastic bag and repeat the previous tests both with the battery in the mouth (to remove light stimulus generated by the LED and control for occipital cortical stimulation) and with rest periods of the battery out of the mouth in the left hand. These battery tests were completed with the devices within a sealed plastic bag to shield the subject from direct contact with the battery and circuit. Overall tests were conducted as given in Table 1.

**Table 1:** Summary of tests related to effects of externally generated magnetic fields Each test completed is described in a tabulated format with test number on the left, description of activities during testing in the middle column, and total test time in the right column Abbreviations: V, voltage; Hz, Hertz

Test	Description	Total Time
1	Subject 1 completed 30 seconds of rest with a 3V circuit in hand followed by 60-second series of a 3V circuit in the mouth with right hand tapping at 2 Hz alternating with 30 seconds of rest with the circuit in hand	210
2	Subject 1 completed 30 seconds of the 6V circuit in hand followed by 60-second series of the 6V circuit in the mouth with 2-Hz right hand tapping alternating with 30 seconds of rest with the circuit in hand	210
3	Subject 2 completed 30 seconds of the 3V circuit with a slow LED in hand followed by 60 seconds of the circuit in the mouth with right hand tapping at 2 Hz alternating with 30 seconds of rest with the 3V circuit in hand	210
4	Subject 2 completed 30 seconds of the 3V circuit with a fast LED in hand followed by 60 seconds of the circuit in the mouth with right hand tapping at 2 Hz alternating with 30 seconds of rest with the 3V circuit in hand	210
5	Subject 2 completed 30 seconds of the 6V circuit with a slow LED in hand followed by 60 seconds of the circuit in the mouth with right hand tapping at 2 Hz alternating with 30 seconds of rest with the 3V circuit in hand	210
6	Subject 2 completed 30 seconds of the 6V circuit with a fast LED in hand followed by 60 seconds of the circuit in the mouth with right hand tapping at 2 Hz alternating with 30 seconds of rest with the 3V circuit in hand	210

Distance testing

Testing was then conducted to investigate the effect of decay of the EMF from a distance with reproduced tapping exercises. Volunteers were asked to lie in the Faraday cage as above and participate in reproduced repeated tapping at 2 Hz. Subjects were asked to alternate between resting and tapping using the above protocol with tapping of the right hand. Sensors were placed within the EMF channel acting as a waveguide as described above and gradually pulled further away from the subject. The sensor was pulled back to designated distances at multiples of 4.5 cm as optimal EMF sensing occurred at 4.5 cm, as previously described [4]. Testing distances occurred at 1 cm, 4.5 cm, 9 cm, 18 cm, and as far back as 63 cm in order to identify if there was a distance wherein Mu-metal shielding would not appropriately exclude external EMF and if the Mu-metal shielding would focus the signal effectively. At 63 cm, the left B319 sensor was pulled back, with the remaining sensors at 4.5 cm, to evaluate differences between distances and maintain the directionality of the left B319 sensor toward the left motor strip. This test at 63 cm was repeated without shielding but within the Faraday cage. The sensors were cushioned for movement and fixed inside the tube to prevent movement artifact. Sensors did not touch hair or skin at 1 cm to account for micromovement. Many subjects participated in these activities at multiple distances and sensor positions for reliability and areas being sensed. Testing terminated at 63 cm due to limitations of the size of the Faraday cage. Tests conducted are given in Table 2.

**Table 2:** Summary of tests related to distance Each test completed is described in a tabulated format with test number on the left, description of activities during testing in the middle column, and total test time in the right column Abbreviations: Hz, Hertz; cm, centimeters; EMF, electromagnetic field

Test	Description	Total Time (seconds)
7	30 seconds of inactivity with 60 seconds of right hand tapping at 2 Hz alternating with 30 seconds of inactivity, with sensors placed 1 cm from the subject	210
8	30 seconds of inactivity with 60 seconds of right hand tapping at 2 Hz alternating with 30 seconds of inactivity, with sensors placed 9 cm from the subject	210
9	30 seconds of inactivity with 60 seconds of right hand tapping at 2 Hz alternating with 30 seconds of inactivity, with sensors placed 4.5 cm from the subject	150
10	30 seconds of inactivity with 90 seconds of right hand tapping at 2 Hz followed by 30 seconds of rest, with sensor B319 placed 63 cm away and the remaining sensors 4.5 cm away	150
11	30 seconds of inactivity with 90 seconds of two-hand tapping at 2 Hz followed by 30 seconds of rest, with sensor B319 placed 63 cm away and the remaining sensors 4.5 cm away	150
12	Subject 1 performing 30 seconds of inactivity with 90 seconds of right hand tapping at 2 Hz followed by 30 seconds of rest, with sensor B319 placed 63 cm away and the remaining sensors 4.5 cm away with the head just underneath the helmet	150
13	Subject 2 performing 30 seconds of inactivity with 90 seconds of right hand tapping at 2 Hz followed by 30 seconds of rest, with sensor B319 placed 63 cm away and the remaining sensors at 4.5 cm away with the head just underneath the helmet	150
14	Subject 3 performing 30 seconds of inactivity with 90 seconds of right hand tapping at 2 Hz followed by 30 seconds of rest, with sensor B319 placed 63 cm away and the remaining sensors 4.5 cm away with the head just underneath the helmet	150
15	Subject performing 30 seconds inactivity with 90 seconds of both hands tapping at 2 Hz followed by 30 seconds of inactivity without the helmet but all sensors remained in a shielded Mu-metal EMF channel tube 18 cm away from the subject	150

## Results

Results of testing were compiled into figures after capturing data in real time through FFT. The FFT data are composite data of 20-second bins of composite summative voltage versus frequency recorded over the length of the test.

Effects of external EMF on cortically generated EMF

**Figure 3:**
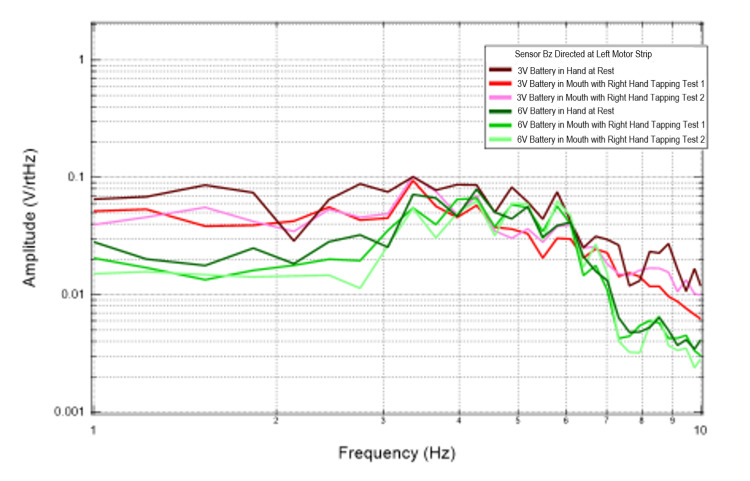
Recordings from sensor Bz when directed at the left motor strip during battery testing Fourier transformed data evaluating effects of an external electromagnetic field measured by sensor Bz on rest and right hand tapping Abbreviations: V, voltage; Hz, Hertz; V/rtHz, voltage divided by square root of Hertz

**Figure 4:**
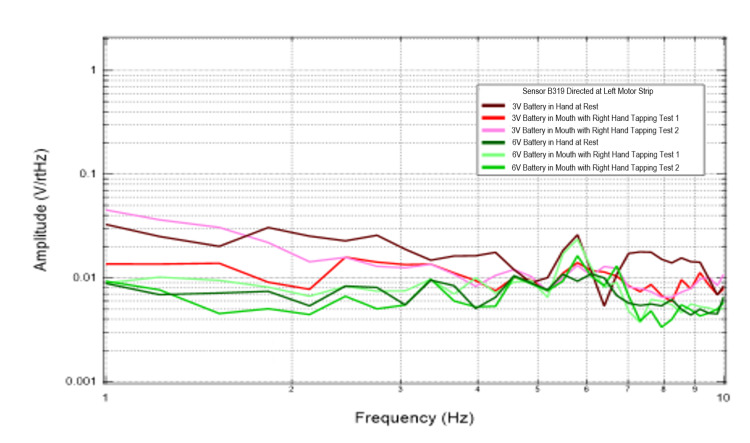
Recordings from sensor 319 when directed at the left motor strip during battery testing Fourier transformed data evaluating effects of an external electromagnetic field measured by sensor B319 on rest and right hand tapping Abbreviations: V, voltage; Hz, Hertz; V/rtHz, voltage divided by square root of Hertz

Additional trials were conducted to evaluate the effects of external EMF on the magnetic field generated by the brain through introduction of 3V and 6V circuits connected to an LED. Figures 3, 4 identify results of this test graphically. When evaluating sensors Bz and B319, which were directed at the left motor strip, the recordings generated at the lowest frequencies identified between 1 and4 Hz appeared to have the greatest summative amplitudes compared to the 6V battery tests. This was seen during rest and tapping. This could be from attenuation of the signal by cortically generated EMF or interference of the EMF waves creating a summatively different dipole.

**Figure 5:**
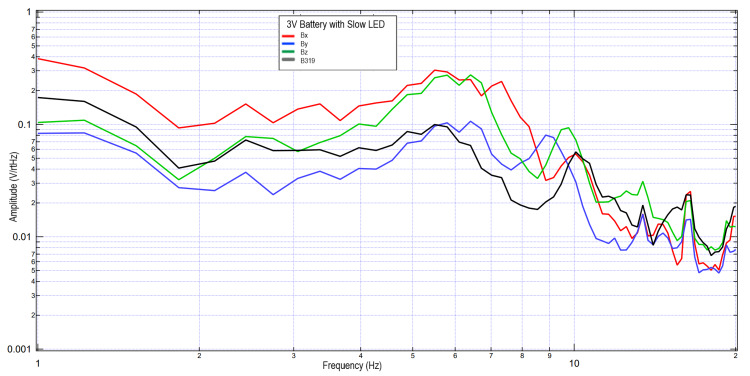
Results of right hand tapping at 2 Hz with Influence of a 3V circuit and a slow LED Fourier transformed data evaluating effects of an external electromagnetic field with a slow-phased LED with a 3V circuit measured during right hand tapping Abbreviations: V, voltage; Hz, Hertz; V/rtHz, voltage divided by square root of Hertz; LED, light-emitting diode

**Figure 6:**
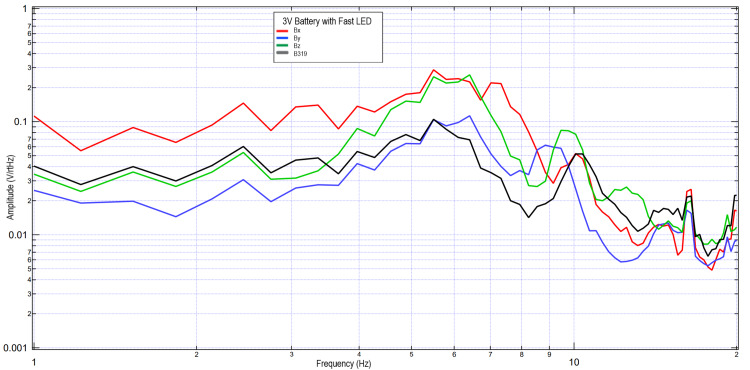
Results of right hand tapping at 2 Hz with influence of a 3V circuit and a fast LED Fourier transformed data evaluating effects of an external electromagnetic field with a fast-phased LED with a 3V circuit measured during right hand tapping Abbreviations: V, voltage; Hz, Hertz; V/rtHz, voltage divided by square root of Hertz; LED, light-emitting diode

**Figure 7:**
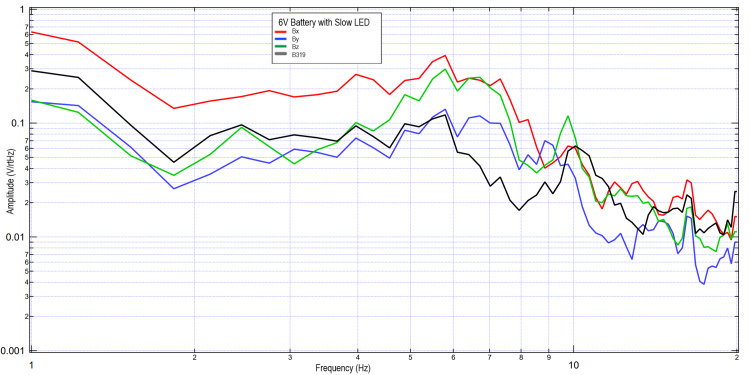
Results of right hand tapping at 2 Hz with influence of a 6V circuit and a slow LED Fourier transformed data evaluating effects of an external electromagnetic field with a slow-phased LED with a 6V circuit measured during right hand tapping Abbreviations: V, voltage; Hz, Hertz; V/rtHz, voltage divided by square root of Hertz; LED, light-emitting diode

**Figure 8:**
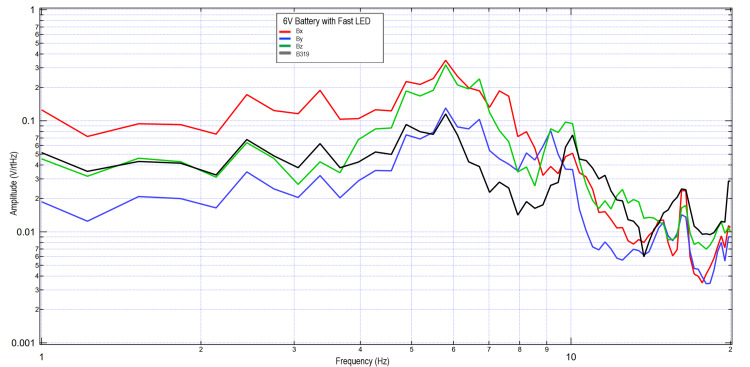
Results of right hand tapping at 2 Hz with influence of a 6V circuit and a fast LED Fourier transformed data evaluating effects of an external electromagnetic field with a fast-phased LED with a 6V circuit measured during right hand tapping Abbreviations: V, voltage; Hz, Hertz; V/rtHz, voltage divided by square root of Hertz; LED, light-emitting diode

A second subject completed similar tests using 3V and 6V circuits. These data were further plotted in Figures 5-8 when investigating the effects of light stimulation on cortical activity. It is seen when evaluating the slow versus fast LED that the morphology of the peaks is different, with increased sharpness peaks with the fast LED compared to the slow LED. This may be due to additional cortical stimulation from changes in light. Evaluating the 3V versus 6V amplitudes did not redemonstrate the pattern seen in Figures 2, 3, which may identify that the changes in EMF may be due to influence from external signal within the helmet and the effects on the EMF dipole or may be from changes from the additional cortical stimulation from light itself shifting amplitudes.

Effects of EMF distances on sensor reading when using an EMF channel

**Figure 9:**
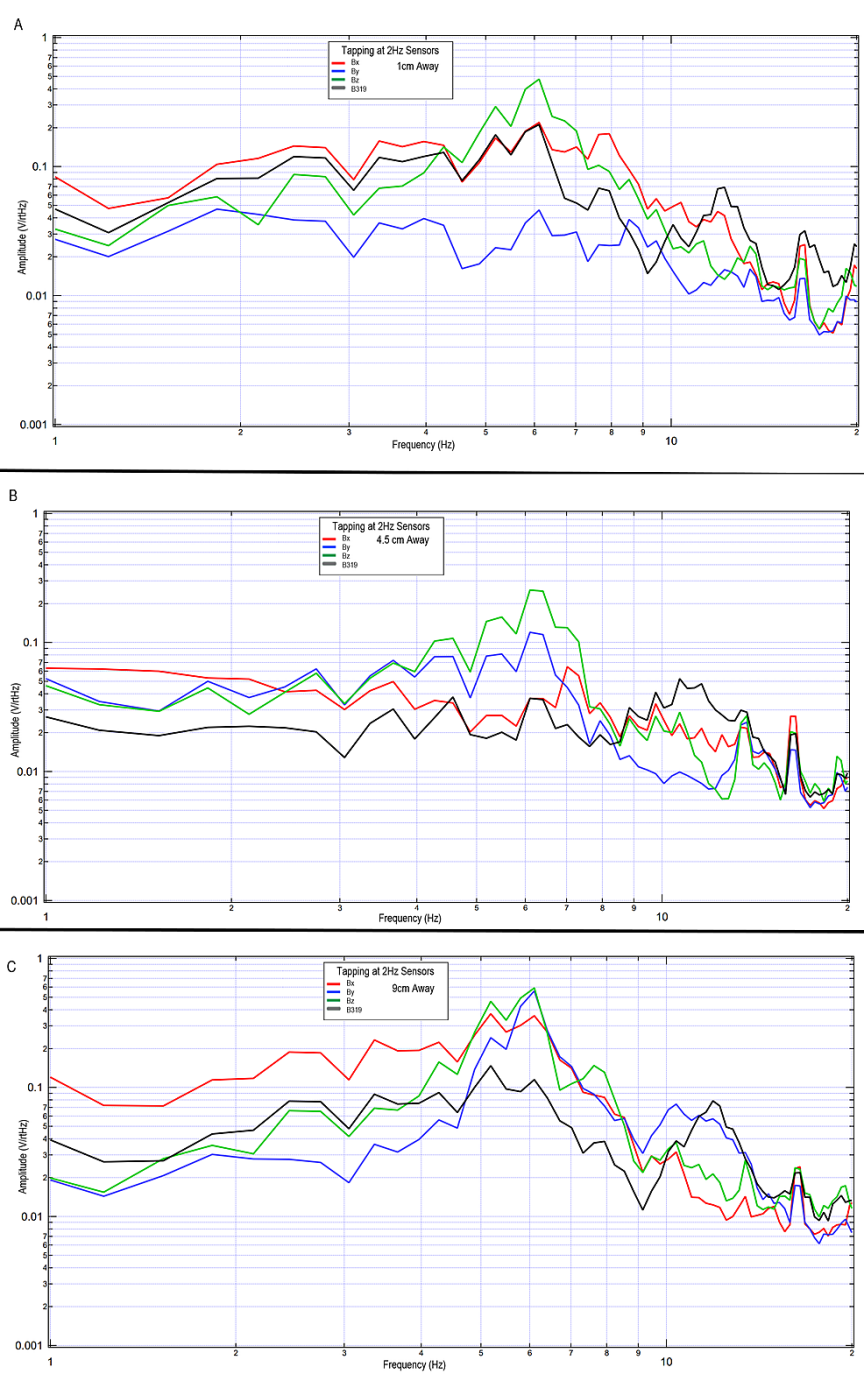
Differing morphologies of waves based on differing sensor distance from the subject within the shielded helmet when tapping at 2 Hz Fourier transformed data evaluating effects of sensor distance away from the subject's scalp on measured electromagnetic field during motor activity Panel A: sensors 1 cm away, Panel B: sensors 4.5 cm away, Panel C: sensors 9 cm away Abbreviations: Hz, Hertz; V/rtHz, voltage divided by square root of Hertz; cm, centimeters

The effects of distance on sensed EMF while a subject completed right hand tapping at 2 Hz using the standardized tapping protocol were further investigated during additional testing. Sensors were placed in standard orientations within the shielded helmet, and the sensors were gradually withdrawn while within an EMF channel wrapped in Mu-metal to act similarly to a waveguide and evaluated 1 cm away from the scalp, 4.5 cm away from the scalp, and 9 cm away from the scalp in initial testing. The results of these tests can be seen in Figure 9. Overall similar locations of peaks are identified; however, there are differing morphologies of the peaks when evaluating differing differences. This may identify areas of decay of the EMF or areas of the EMF where peak dipole is not being identified.

**Figure 10:**
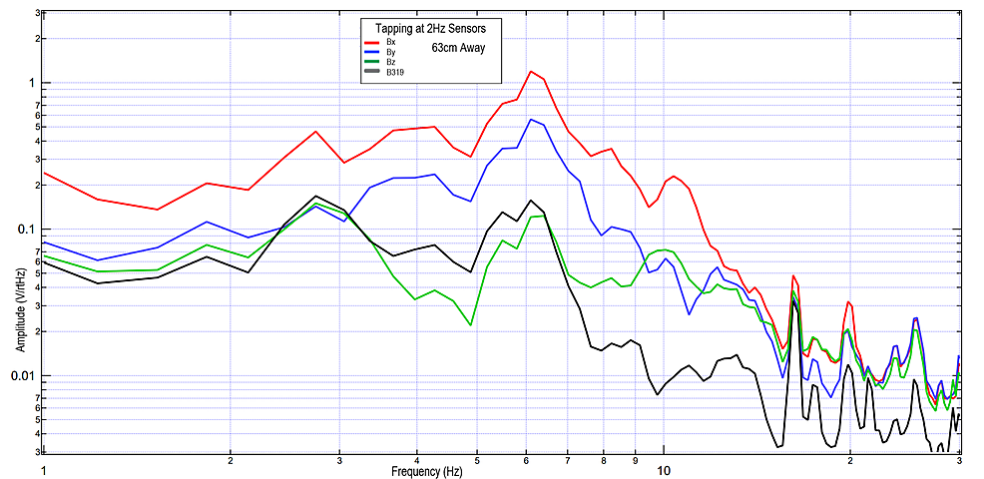
Right hand tapping at 2 Hz with the B319 sensor 63 cm away; sensors Bx, By, and Bz remained 4.5 cm away from the subject Fourier transformed data evaluating effects of maximal technical limit of sensor distance at 63 cm (due to size of the electromagnetic field channel) from the subject's scalp on measured electromagnetic field during motor activity Abbreviations: Hz, Hertz; V/rtHz, voltage divided by square root of Hertz; cm, centimeters

**Figure 11:**
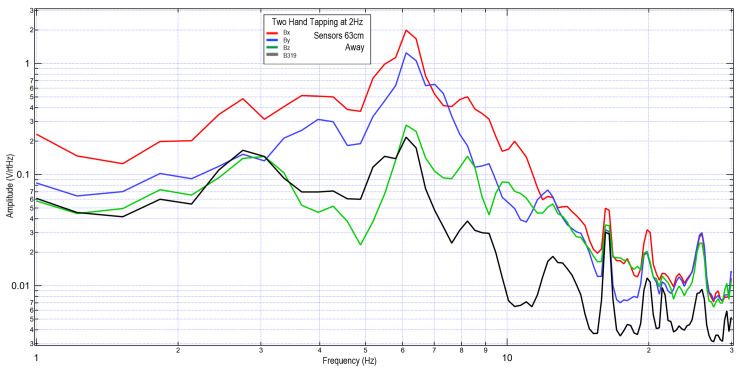
Two-hand tapping at 2 Hz with the B319 sensor 63 cm away; sensors Bx, By, and Bz remained 4.5 cm away from the subject Fourier transformed data evaluating effects of sensor distance on measured electromagnetic field during two-hand tapping exercises. Sensor differences varied in this test, with sensors Bx, By, and Bz remaining 4.5 cm away from the subject while sensor B319 (in black) 63 cm away from the subject. Abbreviations: Hz, Hertz; V/rtHz, voltage divided by square root of Hertz; cm, centimeters

To evaluate the limits of EMF testing using the helmet and EMF channel structure, the B319 sensor was withdrawn to 63 cm near the limit of the EMF channel. The remaining sensors remained at 4.5 cm to act as a control. A subject was asked to tap with the right hand, and tap with both hands at 2 Hz. These results are seen in Figures 10, 11. It was seen that the overall morphology of the waves was similar between all channels; however, the B319 wave was decreased in amplitude relative to the other measured waves compared to the other tests. Overall amplitude was increased with two-hand tapping compared to one-hand tapping, potentially due to additional regions of cortical brain activity.

**Figure 12:**
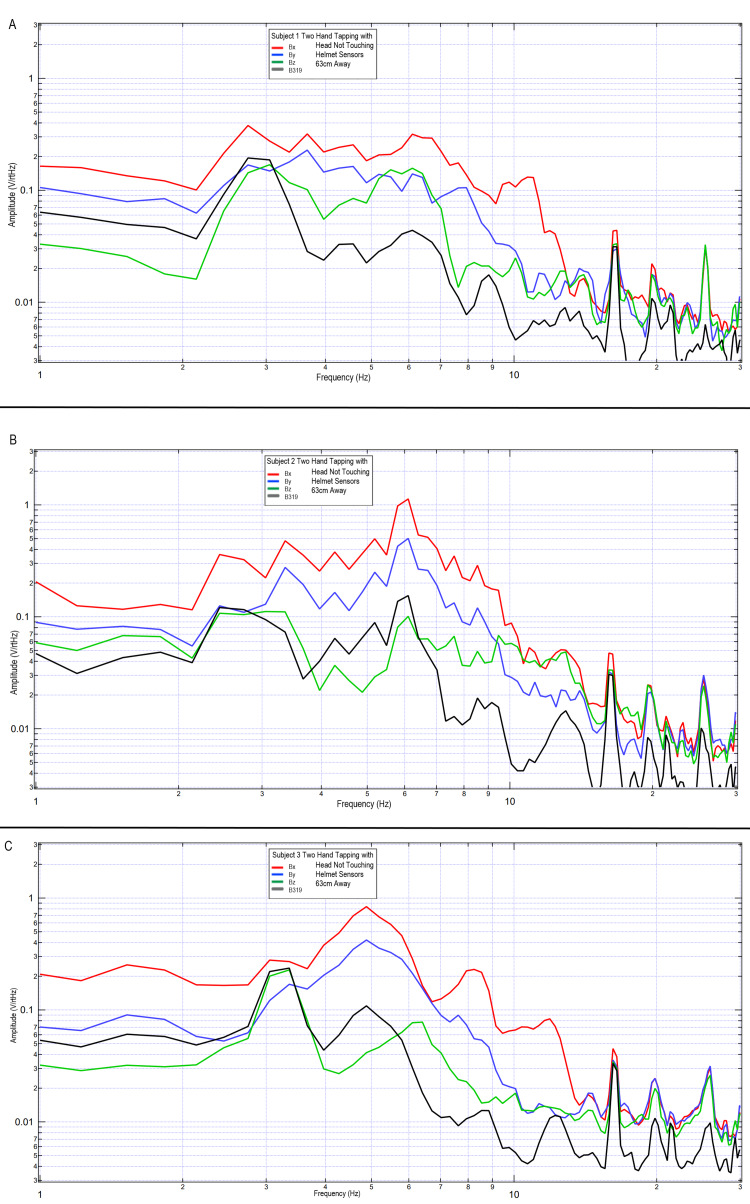
Results of two-hand tapping at 2 Hz among three subjects with sensor B319 at a distance of 63 cm, and sensors Bx, By, and Bz remaining 4.5 cm away Fourier transformed data of effects of sensor distance away from the scalp during two-hand tapping on multiple subjects. Sensors Bx, By, and Bz were maintained at 4.5 cm away from the scalp within the electromagnetic field channel while sensor B319 ( in black) was placed 63 cm away. Panel A: subject 1; Panel B: subject 2; Panel C: subject 3 Abbreviations: Hz, Hertz; V/rtHz, voltage divided by square root of Hertz; cm, centimeters

To evaluate the reproducibility and to evaluate the effects of motion artifact, the previous two-hand tapping trials were repeated using three subjects. These subjects were placed with their head just outside the helmet but with the helmet still surrounding them. Sensor B319 remained 63 cm away, with the Bx, By, and Bz sensors 4.5 cm away from the subjects. Sensors remained within the EMF channel constructs. Subjects tapped at 2 Hz with both right and left hands using the standardized protocol. Results are graphically represented in Figure 12. It was again demonstrated that sensor B319 had a reduction in signal level, seen the most at frequencies greater than 7 Hz, but overall morphology was similar compared to the Bx, By, and Bz sensors.

**Figure 13:**
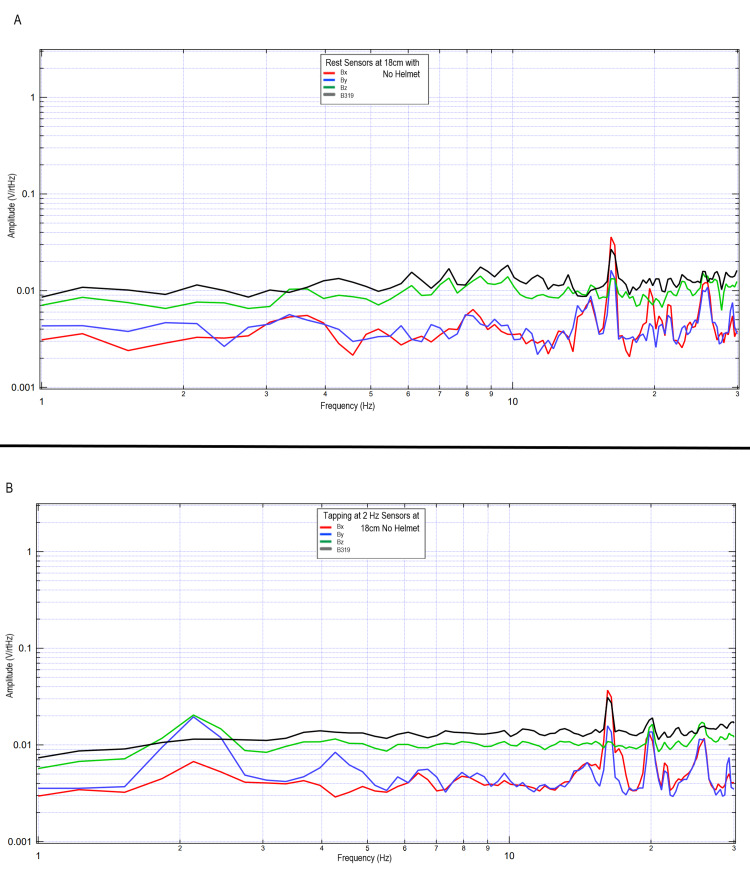
Evaluation of electromagnetic field of a subject participating in two-hand tapping at rest and at 2 Hz without a shielded helmet Fourier transformed data evaluating two-hand tapping and rest without use of a shielded helmet while sensors were placed 18 cm away from the subject Panel A: rest (no helmet used in testing); Panel B: two-hand tapping at 2 Hz (no helmet used in testing) Abbreviations: Hz, Hertz; V/rtHz, voltage divided by square root of Hertz; cm, centimeters

Additional investigations were conducted to evaluate the specific effects of the helmet in regard to shielding effectiveness in the measurement of cortically generated EMF waves. A subject was placed in a PVC cube within a Faraday cage and sensors were placed 18 cm away from the subject fixed within the cube oriented with the same orientations as when in the helmet. The subject did not have the Mu-metal helmet in place; however, the EMF channels remained in place. The subject was asked to tap at 2 Hz using both hands. Results of this trial at rest (upper figure) and with tapping (lower figure) can be seen in Figure 13. The results identify changes in morphology from the previous tests including the 63-cm testing wherein without the helmet shielding, the EMF generated by cortical structures appears to be lost within the intrinsic EMF of the earth and space despite the shielding with a Faraday cage. These changes further identify that there are changes from baseline activity that these sensors are identifying that appear to correlate with cortical activities of the subject.

Overall, it appears that the morphology of the recordings changed as a function of distance away from the subject. This may be due to the effects of proximity on EMF evaluation. In regions of low frequency and higher amplitude at further distances, the amplitude increases may be a function of the effects of the earth's magnetic field on the coil sensors themselves providing some degree of artifact. However, as the inflection points are similar, with similarly identified frequencies, it appears possible that these sensors may have been able to identify some degree of EMF up to 63 cm away when using EMF channels as shielding.

## Discussion

It is well demonstrated that normal physiologic activities of the brain generate an electrical current and a magnetic field [7]. Current technologies allow for measurement of surface potentials with EEG or require super-conduction in the form of magnetoencephalography (MEG) [8]. Although the spatial and temporal mapping yielded by MEG is beneficial, the cost and requirements for super-conduction are limiting in clinical use. Therefore, there was a need to investigate novel, portable, cost-effective, and non-invasive technologies that may measure EMF in real time.

It was previously demonstrated that these novel sensors were able to record EMF from multiple parties and identify changes in EMF during repetitive motor activities [3,4]. Therefore, we recorded brain activity from areas known to be involved in motor movements with sensors placed above the pre-motor region (frontal lobe), motor region (posterior frontal lobe, motor cortex), and association areas (parietal) of the brain. Furthermore, this investigation wished to expand upon this work and evaluate particularities within shielding parameters including investigation of use of a shielded helmet and shielded EMF channel to aid in focusing the EMF similarly to the functions of a wave guide. Furthermore, this trial evaluated effects of endogenous EMF on the measured cortical activities.

It was found that external stimulus through the introduction of a 3V and 6V circuit with an LED may have effects on the measured EMF. This may be due to transmission of the magnetic dipole through the lipid contents of the brain as fatty tissues have been found to act as a media for EMF [9]. Additionally, the external EMF generated by the circuit could have altered the measured dipole in a summative fashion. Further studies will need to be conducted to investigate the effects of external EMF on cortical activities.

It was found that these sensors can measure brain EMF without a large Mu-metal shielded room and without heavy or super-conducting materials and instead with use of alternative smaller shielding technologies. It appeared possible to obtain effective measurements using a portable lightweight helmet. The helmet contained two Mu-metal layers separated by 2.5 cm thought to reflect the signal. There was an inner layer and an outer layer of interlaced copper mesh thought to absorb EMF. Furthermore, each sensor tube was wrapped with Mu-metal foil and housed within a tube in order to channel the EMF. This configuration was used to determine if adequate shielding could take place on the subject’s head and without the need for a Mu-metal external enclosure. When evaluating the use of the helmet in addition to the EMF channels, a degree of the EMF could be measured as far as 63 cm away from the subject. Furthermore, when placed closer to the subject, the generated EMF appeared to have improved resolution, as seen in Figure 9. Specificities of these measurements correlating directly with clinical applications have not been completed yet in regard to sensitivity and specificity; however, it appears that the shielding through use of a helmet and EMF channel may provide appropriate scaffolding in order to measure the generated EMF in a non-contact non-invasive fashion. Furthermore, it is hypothesized that the EMF measured is the EMF through a cylinder defined by the distance away from the subject through the EMF channel. Additional investigation may is needed to identify overlapping areas of sensing and machine learning to aid in localizing through these overlapping cylinders of measurement to generate mapped activity. Also, future studies are needed to correlate the measured EMF with a functional magnetic resonance imaging (MRI) image to correlate recorded measurements and changes with function to provide an additional method of mapping. Furthermore, if EMF can be measured at a distance while using the helmet, this may aid in the design of medical technologies for measurement.

The sensors were also independently wrapped in Mu-metal shielded tube, the EMF channel, but without the described helmet. This was demonstrated in Figure 13. It appears that measuring the EMF activity without the shielded helmet demonstrated decay of the measurements without discernable signal identified. This signal may have been dampened by other signals within the room or may not have been sufficiently identifiable without the helmet. As such, the helmet and EMF channel shielding technologies appeared to be mutually beneficial to both focus the signal and functionally exclude extraneous signals.

When investigating the distance from the subject, there appeared to be the largest voltage per square root of Hertz at 9.0 cm. However, this signal does not contain as many absolute changes in frequency as when the sensor is closer, as demonstrated by the altered peaks in Figure 9. Thus, the spatial resolution appears improved with improved proximity. Furthermore, spatially it seems that the best resolution identified through these trials occurred approximately 4.5 cm away from the scalp surface. The effects of distance may be due to the sensor identifying a more finite region of interest the further away from the subject. Additional possibilities could include some degree of decay of the signal with distance. Further correlation with functional technologies such as MEG may assist in elucidating this further. Should this morphology change be a function of focusing on a smaller region, the more proximally located sensor may identify a larger cone of view of regions generating ionic movement, while when measuring at a distance, the evaluated signal may be a smaller and effectively a focal point. If localization can be effectively conducted using these novel induction sensors and EMF channel and helmet construct, there may be clinical implications in neurology and neurosurgery. This would potentially allow for this helmet to be used in a diagnostic capability once normal and abnormal values have been established. This may yield benefits in identifying epileptiform foci, structural lesions that may alter EMF, or even traumatic brain injury wherein alterations in signaling pathways due to injury may potentially be identified. Future studies may be considered to evaluate normal and abnormal values to establish clinical use.

Limitations

Our study was limited due to a small overall sample size limiting generalizability to larger populations. Furthermore, due to the novel nature of these EMF sensors, helmet, and EMF channel, comparisons with current technologies are limited. Further studies may need to be conducted on larger populations and with comparisons with current technologies such as MEG and functional MRI to evaluate further validity. Additional studies without a shielded room may additionally be conducted to evaluate for the feasibility in measuring the EMF without a shielded room but with the helmet and EMF channel construct to increase access and availability in more traditional clinical contexts for future studies in pathologic conditions. These future studies in clinical populations may allow for the investigation of clinical applicability of these measurements. Further studies are additionally required to evaluate and define "normal" measured EMF values.

## Conclusions

This study demonstrated the possibility of measurement of cortically generated EMF functional information when performing prescribed activities. This activity was measurable at a distance as far as 63 cm but had a wider subset of gathered information and resolution at more proximal distances. Differences within morphology were identified at differing distances potentially related to a focusing effect of the measured EMF at further distances and increased regions of evaluation the more proximal the sensor. Furthermore, it appears that exogenous EMFs may have some effects on measured EMF whether it be through cortically driven attenuation or external influence. Finally, the use of a shielded helmet with a shielded EMF channel appears to be functionally efficient at measuring the cortically generated EMF at a distance without the need for super-conducting equipment in a non-invasive manner in real time.
